# Low-abundant bacteria drive compositional changes in the gut microbiota after dietary alteration

**DOI:** 10.1186/s40168-018-0469-5

**Published:** 2018-05-10

**Authors:** Jacquelynn Benjamino, Stephen Lincoln, Ranjan Srivastava, Joerg Graf

**Affiliations:** 1 Department of Molecular and Cell Biology, University of Connecticut, 91 N. Eagleville Road, U-3125, Storrs, CT 06269 USA; 2 Department of Chemical and Biomolecular Engineering, University of Connecticut, Storrs, CT USA

**Keywords:** Termite microbiota, 16S rRNA gene sequencing, Artificial neural network, Deep learning, Low-abundant drivers

## Abstract

**Background:**

As the importance of beneficial bacteria is better recognized, understanding the dynamics of symbioses becomes increasingly crucial. In many gut symbioses, it is essential to understand whether changes in host diet play a role in the persistence of the bacterial gut community. In this study, termites were fed six dietary sources and the microbial community was monitored over a 49-day period using 16S rRNA gene sequencing. A deep backpropagation artificial neural network (ANN) was used to learn how the six different lignocellulose food sources affected the temporal composition of the hindgut microbiota of the termite as well as taxon-taxon and taxon-substrate interactions.

**Results:**

Shifts in the termite gut microbiota after diet change in each colony were observed using 16S rRNA gene sequencing and beta diversity analyses. The artificial neural network accurately predicted the relative abundances of taxa at random points in the temporal study and showed that low-abundant taxa maintain community driving correlations in the hindgut.

**Conclusions:**

This combinatorial approach utilizing 16S rRNA gene sequencing and deep learning revealed that low-abundant bacteria that often do not belong to the core community are drivers of the termite hindgut bacterial community composition.

**Electronic supplementary material:**

The online version of this article (10.1186/s40168-018-0469-5) contains supplementary material, which is available to authorized users.

## Background

Symbioses are widespread in nature, and beneficial digestive-tract symbioses have been shown to be critical for host health [1]. The benefits and contributions of the gut microbiota include enhancement of digestion efficiency, provision of nutrients and vitamins, and procurement of digestive enzymes [2]. Members of the microbiome can contribute to host health by detoxifying allelochemicals from plants, such as tannins, flavonoids, and alkaloids, along with creating colony resistance against possible pathogens [3]. When the host feeds on a nutrient-poor diet, the reliance on the physiological capabilities of the microbes is even greater. Mammals that feed on a cellulose-rich diet, such as ruminant cows, require a gut bacterial community to generate energy for the host due to the inability of mammals to produce cellulases [4, 5]. In contrast, some insects can produce cellulases and sometimes harbor protist symbionts that are crucial to the breakdown of the wood meal [4]. These insects rely on the bacterial symbionts to provide a source of energy in the form of short-chained fatty acids (SCFAs) and for nutrients that are present at low amounts or are absent in plant food sources, such as nitrogen, amino acids, sterols, and many B vitamins [6, 7].

Insect-feeding habits have been shown to partially dictate the microorganisms present in the gut. Cockroaches fed a low-protein and high-fiber diet showed decreases of *Streptococci* and *Lactobacilli* in their gut, coinciding with the reduction of acetate and lactate production [8]. The gut of the American cockroach, *Periplaneta americana*, is populated by a higher abundance of protozoa when fed a high-cellulose diet [9]. The house cricket, *Acheta domesticus*, shows reduced production of H_2_, CO_2_, and SCFAs when fed a high-protein diet compared to other diets [10]. A comprehensive study on higher termites showed that diet plays a role in shaping the gut microbiota. Bacteria with the ability to degrade cellulose were observed to be present in higher abundances in wood-feeding termites compared to termites fed diets without cellulose [11]. In contrast, termites that feed on humus and soil have a more alkaline gut environment, and bacteria that live in more alkaline environments were shown to be more abundant in these termites [12]. *Reticulitermes flavipes*, the eastern subterranean termite, is a wood-feeding lower termite that harbors protist, bacterial, and archaeal symbionts. The protists are thought to aid in the breakdown of cellulose and lignocellulose, while the bacteria and archaea use the breakdown products to produce nutrients for the symbiotic community and the host [13, 14].

Although microbiomes are being extensively studied, temporal studies are limited in their scope, and predictive in silico modeling of microbiome dynamics is lacking. Only a few studies have attempted to model a microbiome, learning about the dynamics between members of the community and environmental factors [15]. Reasons for the lack of microbiome models include the inherent complexity of most communities, the computational difficulty of modeling highly nonlinear relationships, and the need to account for the effect of many external influences, such as substrate, temperature, pH, and micronutrient concentrations. One of the few studies performed in this area involved using an artificial neural network (ANN) with Bayesian network inference to predict the relative abundance of a microbial taxon in the English Channel as a function of its environment [16]. While this method was successful at modeling how the environment shapes the microbiota, it did not answer the question of how to identify important taxa or environmental factors once the dynamics are learned. Similarly, previous studies observed which organisms in the rhizosphere microbiome are important for disease protection in plants, but there is no current method to determine which taxa or environmental factors influence the growth or decline of these organisms based on a learned model [17]. Combining the qualitative knowledge of the bacterial members of a microbiome with quantitative in silico modeling of microbiomes is key for identifying influential organisms in the microbiome, as well as for learning how members of a microbiome work synergistically or antagonistically.

The hindgut microbiome of the lower termite, *R. flavipes*, is suitable for testing predictive models because of the detailed understanding of the community members. Furthermore, *R. flavipes* is capable of feeding on different types of wood and can be easily maintained in the lab [18]. An important aspect of the hindgut microbiome is that a large proportion of the taxa is consistently present in individual termites [18]. These taxa are considered to be part of the “core” community. In this work, the composition of the *R. flavipes* hindgut microbiota was monitored by sequencing the V4 region of the 16S rRNA gene over time following dietary changes. An algorithm inspired by Larsen et al. [16] used a deep artificial neural network (ANN) to learn the dynamics of the microbiota resulting from external influences, such as changes in the diet, and from changes in the relative abundance of the taxa in the microbiome observed in the 16S rRNA gene sequencing data. The ANN was then trained on this data, and a sensitivity analysis was performed to determine the accuracy of the model. When used in conjunction with microbial community analyses, the ANN-learned dynamics allowed for an in-depth analysis of the microbiome to understand taxon-taxon and taxon-substrate interactions.

## Results

### Effect of dietary changes on the termite hindgut microbiota

The hindgut microbiota of termites supplies the host with energy and nutrients by fermenting the ingested lignocellulose. While it has been shown that this community structure changes when termites are fed different diets [19, 20], it is not known how fast these changes occur. By dividing members from a single colony into different groups, which were provided with different food sources and sampled over a 7-week period, we were able to determine how a change in the diet affected the termite hindgut microbiota.

**Fig. 1:**
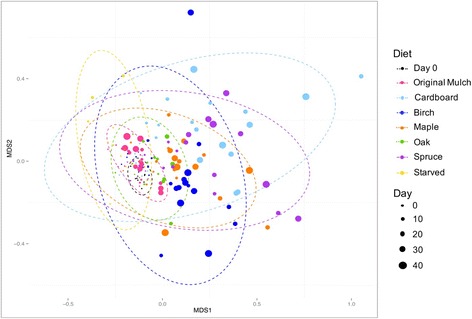
Diet change causes shifts in the hindgut microbiota. An MDS plot using the Bray-Curtis dissimilarity metric shows the shifts in the bacterial community maintained on different diets for 49 days (PERMANOVA, *f* = 4.18, *p* = 0.001). Ellipses show the standard deviation of the mean within each diet

The overall composition of the hindgut community was assessed by determining the Bray-Curtis beta diversity and depicting the resulting values on a multidimensional scaling (MDS) plot (Fig. 1). All day 0 samples were similar to each other. The microbiota of termites that were maintained on original mulch did not exhibit a significant change in the overall composition of the community when compared to day 0 samples (PERMANOVA, *f* = 2.03, *p* = 0.029). The microbiota of termites maintained on all other diets gradually moved away from the day 0 samples over time, which is indicative of a temporal effect of the food source on the hindgut microbiota. For the first 7 days, the communities were similar to day 0, but samples from later dates differed significantly, suggesting that dietary changes affected the composition of the hindgut microbiota (PERMANOVA, *f* = 4.18, *p* = 0.001). This finding suggests that the hindgut microbial community shifted after approximately 7 days of feeding on a new diet.

**Fig. 2:**
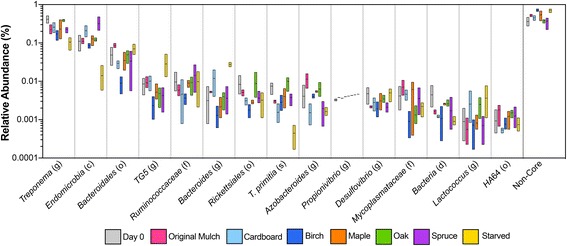
Effect of diet on the core and non-core taxa in the hindgut. The relative abundance of sequences from OTUs present in the core and non-core taxa were calculated for each diet. The boxplot ranges from the minimum to maximum abundance values, with a line at the mean. No diet had a significant effect on sequences from the core taxa as compared to the initial day 0 abundances

Observed differences in a microbial community can be due to instances in which OTUs (operational taxonomic units) are present or absent. Differences may also be explained by a change in the abundance of sequences from any given OTU. The microbes present in the hindgut can be divided into members of the core, which are consistently present, and non-core taxa, which are present intermittently [21, 18]. For this analysis, the sequence counts for each OTU belonging to the same taxon were combined, and the taxonomic abundance values for all time points in each diet were plotted (Fig. 2). *Treponema* and *Endomicrobia* sequences accounted for > 10% of the sequences for all diets, excluding samples from starved termites. Members of these two taxa are known to be associated with the hindgut protists, which decrease in number when the termite is starved, likely leading to a concomitant decrease in their endo- and ectosymbionts [22]. The order *Bacteroidales* accounted for 1–10% of the sequences in all samples, with the lowest abundance detected in samples from birch-fed termites. The remaining taxa were present at abundances of less than 3%, with most accounting for < 1%.

**Fig. 3:**
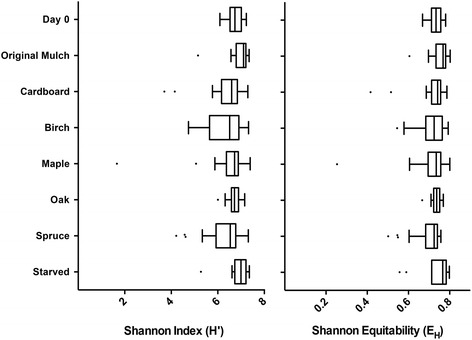
Alpha diversity of the *R. flavipes* hindgut fed multiple diets. The Shannon Index (H′) and Shannon Equitability (EH) metrics were used to calculate the diversity and evenness of the microbiota of the termite hindgut over 49 days when introduced to different diets and plotted using a box and whisker plot. A one-way ANOVA was used to compare the H′ and EH values from each diet to day 0 and showed no significant difference (*p* > 0.05). day 0 (*n* = 9), original mulch (*n* = 27), cardboard (*n* = 26), birch (*n* = 24), maple (*n* = 28), oak (*n* = 17), spruce (*n* = 24), and starved (*n* = 10)

Another characteristic of microbial communities is the diversity of the species present, which can be measured by the Shannon Index (H′), and the evenness of the community, which is measured by equitability (E_H_) metrics (Fig. 3), with a value of zero representing a completely even community and a value of one representing an uneven community. The average H′ and EH values observed were 6.58 and 0.723, respectively. Termites fed birch and spruce showed the most variability within the colony, but there was no significant difference in the richness or evenness among the colonies when compared to day 0 based on a one-way ANOVA analysis.

### Learned microbiome dynamics

The time series data from seven different diets was used at the order level to train an ANN. The robustness of the ANN was evaluated using a tenfold cross validation analysis [23] and by determining the Bray-Curtis similarity. First, a tenfold cross-validation analysis was carried out in which the total data set was randomly portioned into ten equal parts. The ANN was trained on nine parts and validated on the final part. The process was then repeated nine times, with each part being used as the validation set once and the remaining parts used as the training set. The tenfold cross validation analysis yielded an average mean absolute percentage error of 2.5%.

**Fig. 4:**
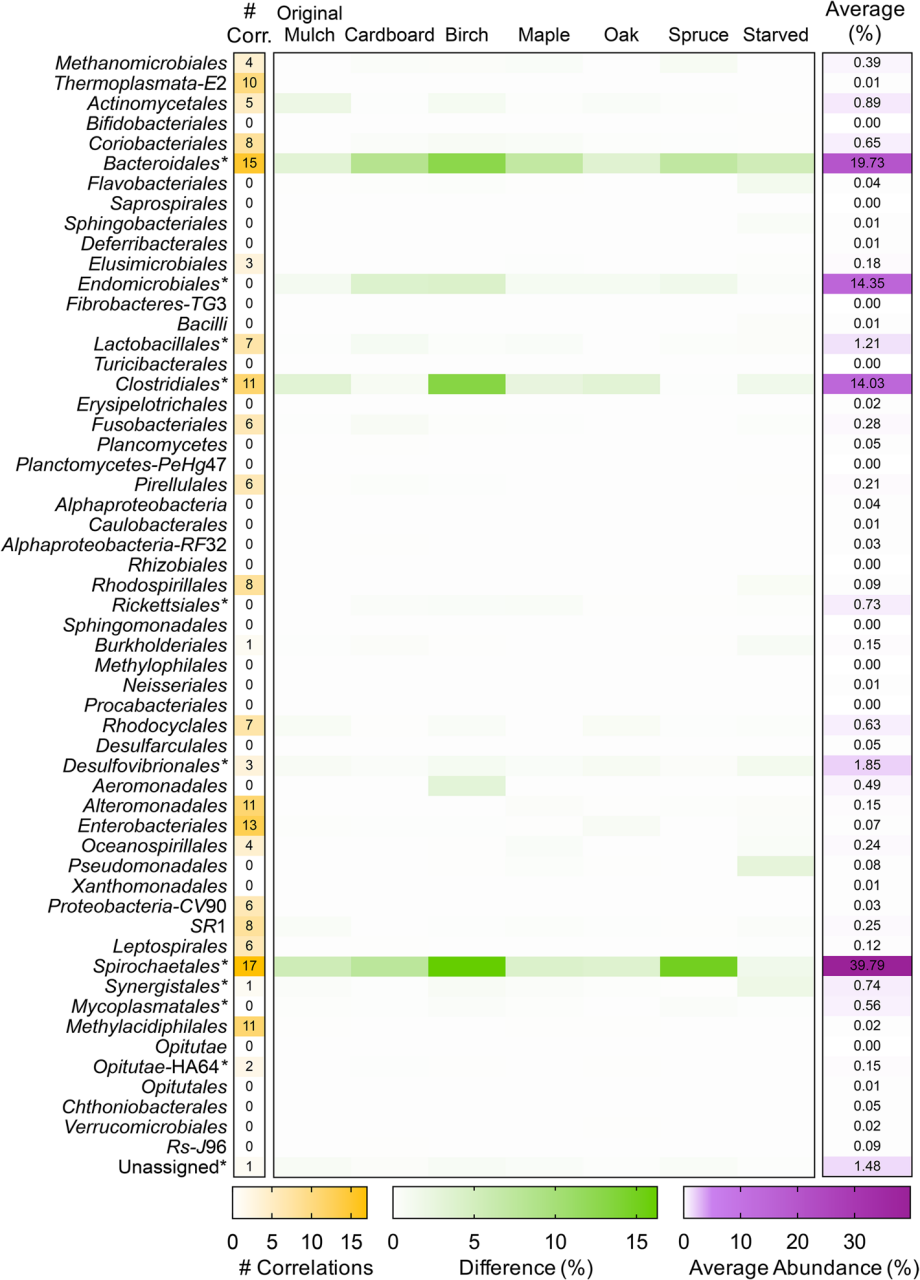
Accuracy of the ANN to predict taxonomic abundances. In training the ANN, one sample per random time point for each diet (along top) was left out and used to test the accuracy of the ANN. The measured abundances of taxa (order) in the samples were compared to the abundances predicted by the ANN. The taxa represented in the core microbiota are denoted by an asterisk, and the average abundances are plotted in the right column (purple). The difference between the actual values and predicted values was calculated and shown in green. The number of significant correlations for each taxon is also shown in the left column (yellow). The ANN was able to predict the taxonomic abundance of each taxon within less than 16% of the measured value. The taxa with the largest differences were present at average abundances of > 14%; therefore, the differences could be due to background noise. The majority of predicted values were < 1% different from the measured values

The ability to predict microbial communities following perturbations is an important facet of microbial ecology. The use of a computationally feasible process, such as ANN, is beneficial for testing and forming hypotheses. To analyze the predictive capability of the network, a single time point was excluded from each of the seven diets, and the ANN was trained on the remaining samples. The termite gut community composition predicted by the neural network was compared to the actual values obtained through sequencing (Fig. 4) at the seven excluded samples. The results showed that the ANN was able to predict the relative abundance of each taxon within 15% of the measured values. The ANN predicted the majority of the taxa within 1% of the measured values. The taxa with higher discrepancies were highly abundant (> 10% abundance). The average abundance of each taxon is also shown (excluding the starved time point). Under these conditions, the observed Bray-Curtis similarity was 0.8681. These results indicated that the network was sufficiently trained and was robust enough to predict the bacterial composition of the microbiome over time.

### Low-abundant taxa shape the gut microbiome

**Fig. 5:**
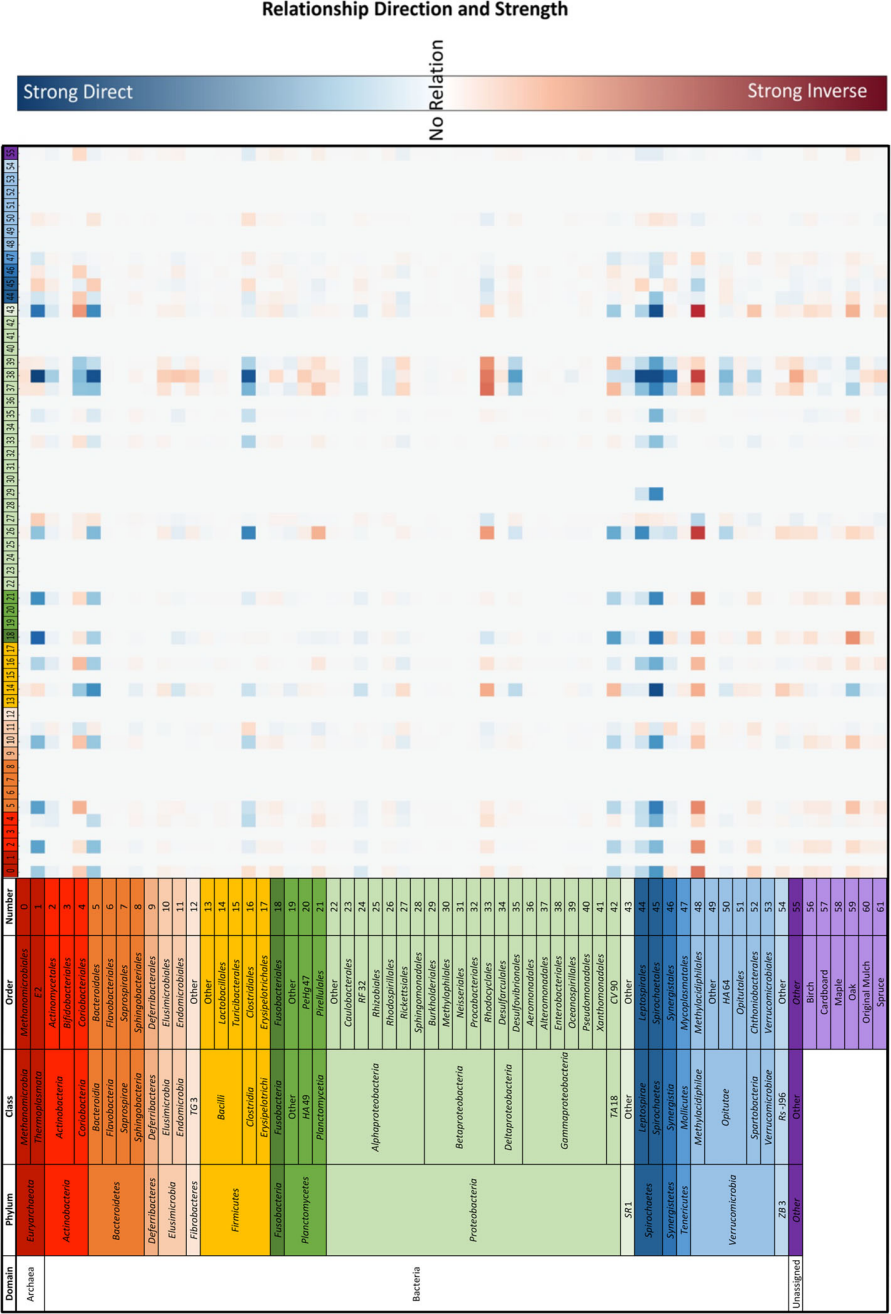
2D heatmap of influences of taxa and substrates on other taxa. The abundance of each taxon/substrate is labeled on the left and corresponding taxa are numbered across the top. Each taxon abundance value was changed by ± 5% (present/absent for substrate), and the effect on the remaining taxa is shown in the heatmap. Direct correlations are shown in blue, and inverse correlations are shown in red

**Fig. 6:**
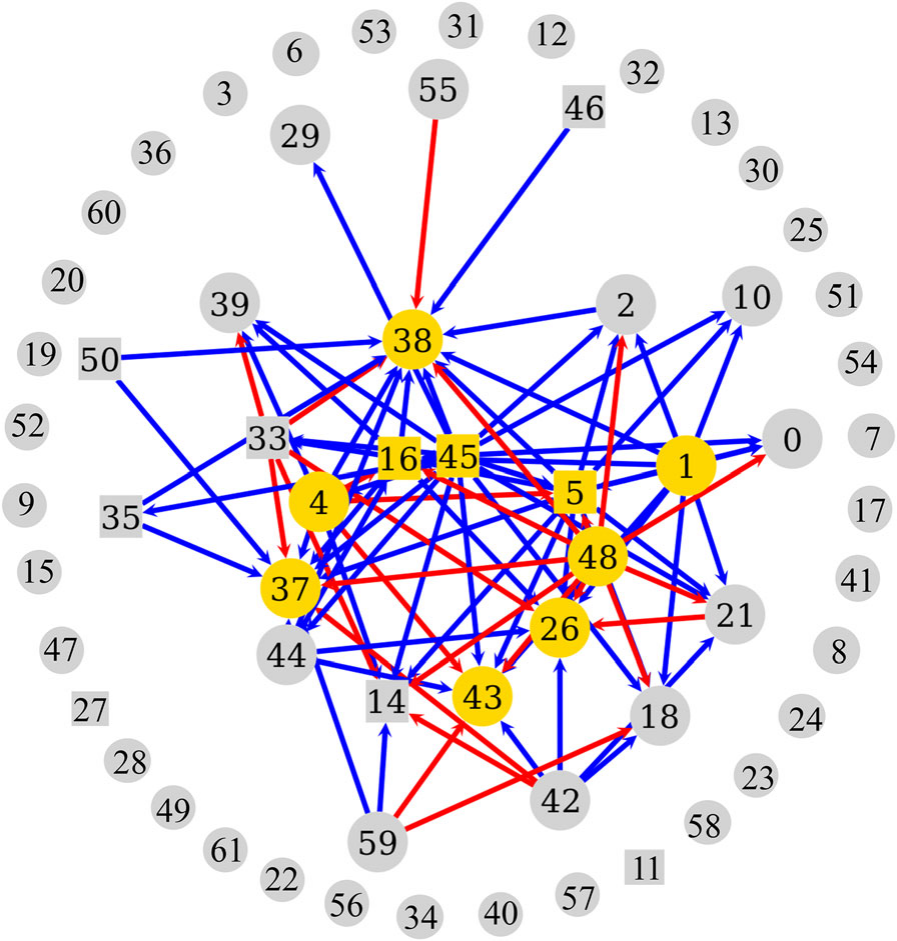
Connectivity network of significant influences of taxa and substrates on other taxa. Each significant correlation observed in the heatmap was plotted using a vertice-edge plot. Direct correlations are shown in blue, and inverse correlations are shown in red. The top ten most abundant taxa are highlighted in yellow. The core members of the microbiota are drawn with a square vertex and the other taxa with a circle vertex

xA 2D heatmap of taxon-taxon and taxon-substrate relationships was created by altering the relative abundance of each taxon/substrate by ± 5% (Fig. 5). As a taxon/substrate (on the left) is changed ± 5%, the shifts in abundance of the other taxa in the community (along the top) are shown in the heatmap, with blue pixels indicating direct relationships and red pixels indicating inverse relationships. The majority of taxa exhibited weak or no relationships, while a number of taxa exhibited strong relationships with a few to many other taxa in the community. The correlations can be divided into outbound, where the taxon affects other taxa, and inbound, where the taxa is affected by another taxon (Fig. 6). All of the 17 correlations associated with *Spirochaetales* were observed to be outbound, suggesting that *Spirochaetales* had the largest effect on the community as a whole. Along with *Spirochaetales*, *Bacteroidales* and *Clostridiales* were abundant taxa with multiple correlations, 15 and 11 respectively. Importantly, the low abundant, highly connected taxa were not detected in reagent control samples, indicating that they are indeed part of the termite microbiome.

**Fig. 7:**
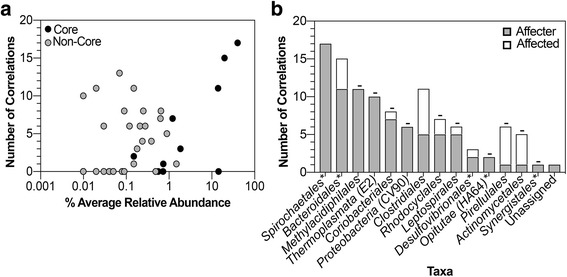
Significantly correlated taxa in the hindgut. **a** A taxon was considered significantly correlated if the value in the heatmap (Fig. 5) was above three standard deviations of the absolute average of the relative-change matrix generated. **b** Core taxa are designated with an asterisk, while taxa < 1% abundance are designated with a minus sign. The majority of significant correlations belong to non-core, low-abundant taxa

The 2D heatmap was used to calculate the taxa and substrates with significant correlations with respect to other taxa (> 3 standard deviations of the absolute average of the % change matrix). Seven core taxa had significant correlations, while 18 non-core taxa had significant correlations (Fig. 7a). Fifteen of the taxa with significant correlations were affecters of the community (Fig. 7b). Bacterial taxa present at relative abundances of greater than 1% contributed to 36 total affecter correlations, while taxa present at a relative abundance of less than 1% accounted for 49 total affecter correlations. Taxa with greater than five affecter correlations included *Spirochaetales*, *Bacteroidales*, *Methylacidiphilales*, *Thermoplasmata* (*E*2), *Coriobacteriales*, *Proteobacteria* (*CV*90), *Clostridiales*, *Rhodocyclales*, and *Leptospirales*. Seven of the nine taxa lie outside of the core, six of them representing less than 1% of the overall community.

## Discussion

Our study revealed that altering the food source of *R. flavipes* affected the overall composition of the hindgut microbiota without affecting the members of the core taxa. The MDS analysis revealed that after 7 days on a new diet, the overall community differed significantly from the community at day 0. This change in the community structure was driven by the less abundant microorganisms that were separate from the core microbiota. In comparison, the control group that was continuously fed mulch was not significantly different from day 0 after 7 days (*f* = 2.03, *p* = 0.029). A previous study fed *R. flavipes* either a wood or a paper substrate, sampled the hindgut microbiota after 7 days, and reported the similarities and differences between the hindgut bacterial communities [19]. They observed that termites from the same colony that were fed different diets were more similar to each other than termites from different colonies that were fed the same diet after 7 days. However, data for later time points were not reported in the study, time points at which changes in the hindgut community were observed in the present study. Our results are similar to those reported from wood-eating cockroaches and higher termites. In wood-eating cockroaches that are closely related to termites, the members of the core microbiota were shown to be stable during dietary changes [24]. In higher termites that do not harbor protist symbionts, it was shown that the dominant members of the hindgut microbiota remained stable and that only less abundant members were affected [12]. It is interesting that these three studies consistently determined that the core was not affected by dietary changes. This highlights the critical contributions of the individual taxa to the overall function of the hindgut microbiota and animal host. The most abundant organisms in a symbiotic habitat have been shown to perform important functions within the environment, essentially securing their constant presence in the environment [25]. A study of the gut microbiota of 37 adults over 5 years showed that approximately 60–70% of the strains remained stable throughout the study. Furthermore, the most stable organisms were also the most abundant, which supports our findings [26]. The compositional stability of the core microbiota is consistent with the idea that the host and its microbes form one functional unit upon which selection acts, as has been proposed in the holobiont theory [27–29]. In our study, mostly non-core members exhibited altered abundances and contributed to shifts in community structure when the food source was altered suggesting that these variable microbes play a critical role in expanding the holobionts capacity to occupy new niches.

The major exception to the stability of the core microbiota occurred in the group that was starved. Starved termites have been shown to lose their symbiotic hindgut protists, which are associated with *Treponema*, belonging to the order *Spirochaetales*, and *Endomicrobia* extracellular and intracellular symbionts, respectively. Thus, the observed decrease in the relative abundances of *Treponema* and *Endomicrobia* was expected. While the *Treponema* exist as both protist symbionts and free-living bacteria, the *Endomicrobia* are strict endosymbionts of the protists. It was therefore not surprising to observe the greater drop in the abundance of *Endomicrobia* in starved termites compared to *Treponema* [30, 31]. It was interesting to note that the *Endomicrobia* were most abundant in the spruce-fed termites, which was also observed in pine-fed termites by Huang et al., suggesting that this substrate may create a hindgut environment that enriches for the protist and *Endomicrobia* populations [20].

The ANN was used to evaluate the effect of a particular taxon or diet on the other taxa, determine the connectivity of a particular taxon to other taxa, and predict the composition of the community at any given point in time. A temporal comparison of the bacterial OTUs from the day 0 gut communities to end-point communities for each diet showed that dietary changes influenced the composition of the hindgut microbiota in *R. flavipes* (Fig. 1). Predictions from the *in silico* model corroborated these results, as the number or strength of correlations between substrate and taxa were greater in each diet (except maple) compared to the number/strength of correlations for the original mulch (diet of day 0 samples) (Fig. 5). Oak was the only substrate with significant correlations to the gut community, suggesting a disturbance to the gut microbes or the need for different metabolic strategies to breakdown its components. When grouped by order, the *in silico* model showed that *Spirochaetales*, the order to which the genus *Treponema* belongs, was the most connected order, with 17 total important correlations (Fig. 7b), while no correlations for *Endomicrobia* were observed. *Treponema* are acetogenic spirochetes that are hypothesized to contribute to the majority of the acetate production in the termite hindgut, which solidifies its importance to the community as acetate is a vital short chain fatty acid (SCFA) to other microbial members and the termite host [32, 33]. Termite gut *Treponema* live associated and disassociated with protists, and *Endomicrobia* are strict endosymbionts of the protists. If the protists and their endosymbionts are required at a constant level for the degradation of the lignocellulose, irrespective of its source, it may be that their abundance is decoupled from auxiliary changes in the community structure.

A powerful aspect of the neural network analysis was that after having trained the algorithm, it could be used to predict future values with relatively good robustness. This suggests that even for a community as complex as that found in the termite hindgut, and with a relatively sparse sampling frequency, the ANN was able to accurately predict the community composition. One limitation of this analysis is that due to complexity of the interactions, the ANN was performed on the level of taxonomic orders. It would be interesting to perform and evaluate the accuracy of this analysis on data from other time series, including humans. If future implementations are able to use lower taxonomic levels, the ANN could prove to be an important predictor of an unbalanced microbial community or dysbiotic state before it occurs. Understanding the effect of diet on a microbial population is valuable because it provides insight into the dynamics of the symbiotic niche. In a laboratory setting, it is necessary to consider the biological effect diet has on a host organism and its symbiotic bacteria. The ability to predict the taxonomic composition of a community is beneficial for forming hypotheses about an environment and can provide insight into the community dynamics within that environment.

Since the start of the high-throughput 16S sequencing revolution, scientists have reported on microbiomes, often focusing on the more abundant taxa or grouping the populations into phylotypes [34]. Many microbial community surveys draw focus to abundant organisms in order to generate conclusions on impact and importance of microbes. In order to view these bacterial populations in numerous samples, it has been standard protocol to show organisms at abundances greater than 1% [35–37], or group the low-abundant organisms into an “other” category [9, 38]. While this is a widely accepted method of determining correlations between healthy and diseased states and reporting microbial communities in general, researchers may be missing key organisms in the low-abundant population. The importance of low abundant organisms has been reported in other systems, such as *Desulfosporinus* in peatlands. Pester et al. reported finding *Desulfosporinus* at 0.006% of the 16S rRNA reads in peatland communities and determined that this small fraction of the community contributes significantly to the overall sulfate reduction [39]. Sogin et al. reports the importance of the “rare biosphere” in marine environments and explains the contribution of these low members to diversity and the gene pool [40]. Low-abundant microbes may contain a pool of genes, that under specific conditions become activated and carryout metabolic processes important to the overall community [41]. For example, the removal of rare microbes from freshwater samples resulted in the reduced ability of the microbes to neutralize toxins and pollutants [42], suggesting that the rare microbes can perform critical functions in an unfavorable environment. While some microbes are rare, they can contribute to the complete metabolic potential of the community if they are highly active, enhance or trigger the metabolic activity of more dominant members, or contain enzymes needed for complex metabolic processes that are not found in the dominant members [41, 43]. In this study, only four core taxa had greater than five significant correlations, while 13 non-core taxa had greater than five significant correlations (Fig. 7). Only five of the 15 taxa with significant correlations were abundant at greater than 1% of the community. This suggests that although the core and highly abundant taxa perform important functions for the community and are conserved throughout diet changes and time, the non-core and less abundant taxa may play important roles in shaping the gut community.

## Conclusion

Gut microbiome dynamics is an important but challenging topic due to the taxonomic complexity of the community and wide range in abundance of its members. Using the complex microbiome of termites as a test case, we have developed a method using deep learning artificial neural networks for temporal taxon prediction based on environmental conditions and the original state of the microbiome. This method also allows the detection of connectivity of taxa with other taxa based on changes in the abundance of microbes during environmental changes. This deep learning approach revealed that low-abundant bacteria that often do not belong to the core community are drivers of the termite hindgut bacterial community composition.

## Methods

### Experimental design and maintenance

The *R. flavipes* termites were purchased from Connecticut Valley Biological Supply Co. in Southampton, MA and initially maintained on the mulch they were shipped with. These mulch-fed termites were separated into colonies that received distinct diets. The colonies were kept in plastic containers with autoclaved sand and food. Termite colonies were maintained in a dark cabinet at room temperature (~ 23 °C) and kept moist with autoclaved water. The samples used in this study were all from the worker caste.

Colonies were fed either mulch (never changed from original food source); wood from spruce, oak, maple, or birch; or cardboard, while one colony was starved. Termites were sampled on the day of arrival (day 0) and on days 1, 2, 3, 7, 14, 21, 28, 35, 42, and 49 after arrival. Five termites were sampled from each diet source for each time point (three per diet and time point were sequenced, except for day 0). The starved colony was sampled through day 21, and the oak-fed colony was sampled through day 28, both due to the lack of termites available in the colony. The samples from day 0 were previously published from Benjamino and Graf [18]. The wood used was non-treated firewood, the cardboard was from shipping boxes, and the original mulch used was the material shipped with the termites from CT Valley Biological Supply Co. Termite DNA was used for cytochrome C oxidase II gene (COII) sequencing to ensure the termites were *Reticulitermes flavipes*. Primers used for COII sequencing were a modified A-tLEU (5′-CAGATAAGTGCATTGGATTT-3′) and B-tLYS (5′-GTTTAAGAGACCAGTACTTG-3′) from Liu and Beckenbach [44] and previously reported in Benjamino and Graf [18].

### Sample collection and DNA isolation

Hindguts were removed from the termite and separated from the foregut/midgut and rectum. Single hindguts were collected in 1X TE buffer (10 mM Tris-HCl, 1 mM EDTA, pH 8.0). DNA was isolated immediately after collection using a modified (500 μL starting lysis buffer, elution in 30 μL AE buffer) RBB+C isolation protocol as described by Yu and Morrison [18, 45]. During each set of DNA isolations, a reagent-only control was processed and sequenced to test for reagent contamination (ex. Neg.7 for day 7 isolations) (Additional file 1).

### PCR amplification and library preparation

Hindgut samples were amplified using the V4 hypervariable region of the 16S rRNA gene using primers developed by Carporaso et al. [46]. PCR reactions included Phusion High-Fidelity PCR Master Mix with HF Buffer (50% of total volume), 10 μM forward and reverse primers (3% each of total reaction volume), ~ 10 ng DNA, and molecular biology grade nuclease-free water to the final volume [47]. All reactions were amplified in triplicate using the following parameters: 94 °C for 3 min, followed by 30 cycles of 94 °C (45 s), 50 °C (60 s), and 72 °C (90 s), with a final extension of 72 °C for 10 min [46].

Amplicons were purified and size selected using the GeneRead™ Size Selection Kit by Qiagen© to select for 400-bp amplicons according to the manufacturer’s protocol. Samples were then quantified using a Qubit® dsDNA HS Assay and diluted to 4 nM. All samples were pooled in equimolar amounts for sequencing. A mock community was also prepared and sequenced alongside these samples and has been previously published by Nelson et al. [48].

### Sequencing and data processing

Samples were sequenced using an Illumina MiSeq with custom sequencing primers added to the reagent cartridge [46] and sequenced 2 × 250bp. Output reads from the MiSeq were merged to create single reads spanning the entire 254 bp length of the V4 hypervariable region using SeqPrep (https://github.com/jstjohn/SeqPrep), and the PhiX control reads were removed by mapping to the PhiX genome [48]. Data analysis was performed on high-quality reads (Q30 or greater) using Qiime [46]. Operational taxonomic units (OTUs) were determined by clustering reads to the V4 hypervariable region of the DictDb 16S rRNA reference dataset at a 97% identity level [12, 18]. Reads that failed to cluster to the DictDb reference were clustered to the Greengenes reference 16S reference dataset (2013-08 release) at a 97% identity, and then de novo OTU clustering was performed on reads that failed to cluster to a reference [49]. The dataset was checked for chimeras and filtered to remove singleton and doubleton OTUs and then OTUs present at less than 0.0005% [48, 50].

### Sequence analysis

After quality filtering and rarifying to 18,000 sequences per sample, alpha diversity (Shannon Index and Equitability) [51] and the Bray-Curtis beta diversity metric [52] were performed using Qiime 1.9. The OTU table and rarified taxonomy table can be found in Additional files 2 and 3. The Shannon Index and Equitability were graphed using GraphPad Prism version 6.0f for Mac OSX (GraphPad Software, San Diego, CA, USA, www.graphpad.com), and a one-way ANOVA with Bonferroni post-test analysis was performed for each. An MDS plot using the Bray-Curtis metric was created in R 3.2.0 [53, 54]. The PERMANOVA statistical analysis was performed to determine the significance of microbial community differences among the different food sources and temporally [55]. The test used the Bray-Curtis dissimilarity matrix as the input and was performed over 999 permutations and returned a Pseudo-F (f) statistic along with a *p* value (*p*). Each test compared the day 0 samples to the last 2 days of samples in other diets.

Taxonomic abundance data was calculated using the percent abundance of OTUs present in the core microbiota. The relative abundance of each taxon, along with the non-core taxa, was combined for each diet and presented with the mean abundance of the temporal data. The non-core abundances were calculated by combining the remaining OTUs that were not present in the core.

### Artificial neural network

**Fig. 8:**
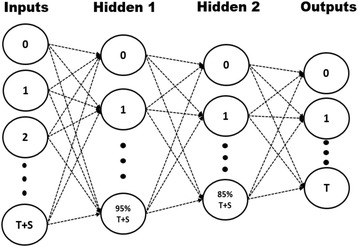
Topology of the ANN used to train on sequenced data. The number of input nodes was set to the number of taxonomic orders (T) plus the number of substrates (S) (70 total). The number of nodes in the first hidden layer was set to 95% of the total input nodes, whereas the number of nodes in the second hidden layer was set to the 85%. The number of output nodes was set to the number of taxonomic orders, as the goal of the network was to predict relative abundance changes over time for each taxon. The arrangement of taxonomic orders remained constant for each CSV file

**Fig. 9:**
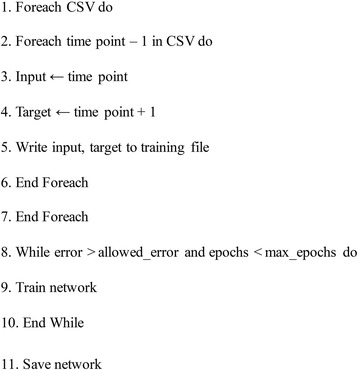
Algorithm for training the ANN

The relative abundance of each OTU was grouped by taxonomic order, as grouping by species for learning microbiome dynamics introduced a significant amount of noise and error to the algorithm. A deep backpropagation artificial neural network (ANN) was created using fast artificial neural network (FANN) [56] with a network topology as shown in Fig. 8. Two hidden layers were utilized due to the ability of deep learning neural networks to learn representations of data with multiple levels of abstraction, such as taxon-taxon interactions and taxon-substrate interactions. In addition, deep neural networks have been shown to implement extremely intricate functions of its inputs that are simultaneously sensitive to minute details and insensitive to large irrelevant variations [57]. The number of nodes in each hidden layer was determined by keeping the number of nodes in each hidden layer close to the number of nodes in the input and output layer as a general rule. In addition, multiple cross validations were performed with different percentages of nodes in each hidden layer with respect to the number of nodes in the input layer. The goal of training the ANN was to learn dynamics of the microbiota based on substrate provided to the colony and the influence of other community members. The network was trained using the relative abundance of each order and the presence or lack of substrate given at a time period *t* for the input nodes. The output represented the relative abundances of each order for the time period *t + 1*. The general algorithm is shown in Fig. 9. Since there was no target for the last time point (day 49), the last time point was never used as an input to the ANN. In addition, one random time point from each comma-separated value (CSV) (7 in total) was left out of training and used for testing the ANN.

The network used the standard backpropagation algorithm native to FANN to train on the dataset until the error threshold was met or the maximum number of epochs had passed. The activation function used in the first hidden layer was the hyperbolic tangent function (*tanh*), while the second hidden layer used the sigmoid function,

1 $$ S(x)=\frac{1}{1+{e}^{-x}} $$

Using an antisymmetric activation function has been shown to improve convergence for more connected networks than an asymmetric activation function, so *tanh* was chosen as the first hidden layer’s activation function [58]. However, the output of the ANN is the relative abundance of a taxon for each output node, which must be in the bounds [0,1]; therefore, the sigmoid function was used for the second hidden layer’s activation function.

After each epoch of training, the mean standard error or MSE was returned. If the error was under the error threshold, training was halted and the network was saved. The network was then tested using the seven time points left out of the training dataset. After testing, the ANN was subject to sensitivity analysis using a test time point from day 0. The parameters of the neural network are shown in Table 1.

**Table 1:** ANN training parameters

Input nodes	62
Hidden 1 nodes	59
Activation 1	Hyperbolic tangent
Hidden 2 nodes	53
Activation 2	Sigmoid
Output nodes	56
Error threshold	10^−5^
Momentum	0.65
Learning rate	0.15
Max epochs	20,000

### Microbiome dynamics analysis

Once the network was sufficiently trained, a sensitivity analysis was performed on the ANN to determine how each taxon changes over time in response to a change in another taxon or substrate. One relative taxon abundance time point for each substrate was kept from the ANN training set and used to test the prediction capabilities of the ANN. Since each input node was representative of a taxon or substrate and each output node was representative of the predicted relative abundance for a given taxon at the next time point, changing each input node individually and comparing the predicted outputs to the original outputs would allow us to discover the learned dynamics. Therefore, each input node was varied independently 100 times to a value ± 5% of the original input node’s value. The ANN was then run for each new value of the input node while holding the other input values constant. After each run of the ANN at the new input node value, the new outputs, or predicted relative abundances of each taxon were recorded and compared to the original output. The percent change of each output node was compared to the percent change of the input node being varied using the following equation:

2 $$ \mathrm{Relative}\ \mathrm{change}=\left(\frac{\left(\mathrm{new}\ \mathrm{output}-\mathrm{original}\ \mathrm{output}\right)}{\mathrm{original}\ \mathrm{output}}\div \frac{\left(\mathrm{new}\ \mathrm{input}-\mathrm{original}\ \mathrm{input}\right)}{\mathrm{original}\ \mathrm{input}}\right)\times 100\% $$

**Fig. 10:**
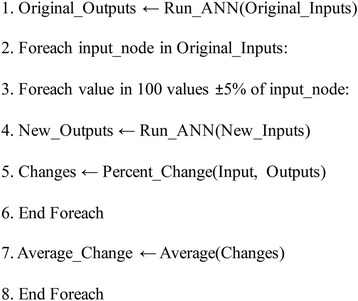
Algorithm for sensitivity analysis

This was repeated for each new value of the input node being tested. After all new values of the input node were tested, the average change of each output node was taken, which showed how each taxon (output node) changed with respect to a change in a certain taxon or substrate (input node). This was repeated for every input node to determine how the relative abundance of each taxon changed with respect to changes in a given taxon or substrate. The general algorithm is shown in Fig. 9. The end result of the sensitivity analysis was a matrix of relative change values (Fig. 10). In other words, each row of the matrix is representative of a taxon or substrate, or an input node of the ANN. Each column is representative of a taxon, or an output node of the ANN. Each value in the matrix is the average relative change of the output node (column) with respect to the input node (row).

A 2D influence heatmap was generated using Matplotlib in Python [59]. The heatmap displays the magnitude and direction (direct/inverse) of each relationship between each input node (taxon/substrate) and output node (taxon). A connectivity network was also generated using the graph-tool library in Python [60]. The connectivity network was constructed using a vertices-edge plot where each vertex was a node in the ANN or a taxon/substrate. An edge was drawn between two vertices if the value in the change matrix was more than three standard deviations above the absolute value of the average of the whole array. The top ten most connected vertices were highlighted and returned from the connectivity network.

## Additional files

Additional file 1: Control samples for 16S rRNA gene sequencing. A Qiime generated OTU table for the positive and negative controls used in the 16S rRNA gene sequencing portion of the manuscript. (XLSX 98 kb)

Additional file 2: 16S rRNA gene sequencing OTU table. The Qiime generated OTU table for the samples used in this study. (XLSX 663 kb)

Additional file 3: Complete taxonomy table. Rarified (18,000 sequences) taxonomy table used in this study. (XLSX 219 kb)

## Abbreviations

ANN: Artificial neural network
ANOVA: Analysis of variance
COII: Cytochrome C oxidase II gene
CSV: Comma-separated value
FANN: Fast artificial neural network
MDS: Multidimensional scaling
MSE: Mean standard error
OTU: Operational taxonomic unit
SCFA: Short-chained fatty acids
